# MAPping tubulin mutations

**DOI:** 10.3389/fcell.2023.1136699

**Published:** 2023-02-15

**Authors:** Thomas D. Cushion, Ines Leca, David A. Keays

**Affiliations:** ^1^ Department of Physiology, Development and Neuroscience, University of Cambridge, Cambridge, United Kingdom; ^2^ Research Institute of Molecular Pathology (IMP), Vienna Biocenter (VBC), Vienna, Austria; ^3^ Division of Neurobiology, Department Biology II, Ludwig-Maximilians-University Munich, Munich, Germany

**Keywords:** microtubules, tubulinopathies, dynein, kinesin, disease, microtubule-associated protein

## Abstract

Microtubules are filamentous structures that play a critical role in a diverse array of cellular functions including, mitosis, nuclear translocation, trafficking of organelles and cell shape. They are composed of α/β-tubulin heterodimers which are encoded by a large multigene family that has been implicated in an umbrella of disease states collectively known as the tubulinopathies. *De novo* mutations in different tubulin genes are known to cause lissencephaly, microcephaly, polymicrogyria, motor neuron disease, and female infertility. The diverse clinical features associated with these maladies have been attributed to the expression pattern of individual tubulin genes, as well as their distinct Functional repertoire. Recent studies, however, have highlighted the impact of tubulin mutations on microtubule-associated proteins (MAPs). MAPs can be classified according to their effect on microtubules and include polymer stabilizers (e.g., tau, MAP2, doublecortin), destabilizers (e.g., spastin, katanin), plus-end binding proteins (e.g., EB1-3, XMAP215, CLASPs) and motor proteins (e.g., dyneins, kinesins). In this review we analyse mutation-specific disease mechanisms that influence MAP binding and their phenotypic consequences, and discuss methods by which we can exploit genetic variation to identify novel MAPs.

## Introduction

### Microtubules

Microtubules are large polymers formed by repeats of α- and β-tubulin heterodimers. Tubulin heterodimers fold *via* a highly conserved and complex pathway involving chaperones, chaperonins and other co-factors (Lewis et al., 1997). Once folded, the α- and β-subunits bind a molecule of guanosine triphosphate (GTP) each, at two conserved structural motifs: the non-exchangeable site and the exchangeable site (Nogales et al., 1998; Lowe et al., 2001). Heterodimers assemble longitudinally into protofilaments, arranged uniformly with β-tubulin exposed at the growing tip, with 13 protofilaments associating laterally to form a hollow, cylindrical structure (Nogales, 2001). Microtubules cycle between periods of steady growth (polymerisation) and rapid collapse (de-polymerisation or “catastrophe”), by the addition or loss of tubulin heterodimers (Mitchison and Kirschner, 1984). This behaviour is utilised by every mammalian cell to perform a range of functions, including the control of cell morphology, cell motility, intracellular transport, and cell division. To accurately perform such a wide repertoire of tasks, microtubules are subject to regulation on multiple levels. Whilst they are often depicted as homogenous chains of α/β-tubulin heterodimers, microtubules can be constituted by a variety of similar yet subtly distinct α- and β-tubulin isotypes, each compatible with the structure of the microtubule polymer lattice. These tubulin isotypes are encoded for by different tubulin genes distributed across the human genome: eight α- and nine β-tubulins (Breuss and Keays, 2014).

Each of these tubulin isotypes has a unique expression pattern (Leandro-García et al., 2010). For instance, TUBB3 is predominantly found in post-mitotic neurons, TUBA8 in muscles and the testes (Braun et al., 2010), TUBB1 in haemopoietic cells (Leandro-García et al., 2010), and TUBB8 in oocytes (Feng et al., 2016). The consequence of this variation in expression is that microtubules in different cell types consist of a different blend of tubulin heterodimers. This is relevant because it confers different properties on those microtubules, enables different microtubule-associated proteins to bind, and it results in different disease states when they are mutated. While tubulin isotypes share a high degree of sequence homology, they exhibit notable divergence in the unstructured carboxy-terminal tail (CTT) which extends outwards and away from the microtubule wall and into the cell cytoplasm (Nogales et al., 1998). These CTTs are predicted to play an important role in many respects of microtubule biology. Importantly, they are site of multiple reversible post-translational modifications including de-tyrosination, glutamylation and glycylation (Janke and Bulinski, 2011). Despite emerging evidence that CTTs might play an unexpected role modulating microtubule polymerisation dynamics (Parker et al., 2018; Chen et al., 2021), it is their relative accessibility at the polymer exterior that are thought to be critical to the function of another key regulator of microtubule behaviour, the wide range of microtubule-associated proteins (MAPs).

### microtubule-associated proteins

Microtubule-associated proteins were originally defined as those proteins that purified with microtubules from brain extracts (Sloboda et al., 1975). With the passage of time and the development of various methods, this criterion has been refined. In addition to co-sedimenting with microtubules, MAPs should co-localize with microtubules by immunofluorescence in cultured cells and their staining pattern should become dispersed upon addition of depolymerizing drugs (Huber et al., 1985; Bodakuntla et al., 2019). MAPs can be further categorized based on their function and/or localization on microtubules (Tortosa et al., 2016; Tortosa et al., 2017; Goodson and Jonasson, 2018; Bodakuntla et al., 2019). Motor proteins (e.g., dyneins, kinesins) are MAPs responsible for generating cellular forces and for intracellular transport. Some MAPs contribute to microtubule nucleation (e.g., doublecortin) while others promote catastrophe by depolymerization or severing (e.g., spastin, katanin). “+TIP” binding proteins (e.g., EB1-3, XMAP215, CLASPs) and minus-end binding proteins (e.g., CAMSAP1-3) bind to the plus- and minus-ends of microtubules respectively, whilst structural MAPs bind along the lateral-wall (lattice) of microtubules, acting as cross-linkers with intermediate filaments and the actin cytoskeleton (e.g., MACF1, MACF2) (Hendershott and Vale, 2014; Tortosa et al., 2016). Some authors also consider tubulin-modifying enzymes as MAPs, since they necessarily interact with microtubules to deposit specific PTMs (Kapitein and Hoogenraad, 2015; Tortosa et al., 2016), as well as several metabolic enzymes that have been shown to bind microtubules (Walsh et al., 1989; Lloyd and Hardin, 1999). In addition, there are MAPs that are recruited to the microtubules indirectly, *via* other proteins that bind to microtubules. Examples include the phosphatase, PP1, recruited to microtubule polymers by tau, and a group of kinases, MAST1-4, that preferentially colocalize with microtubules in the presence of other MAPs (Walden and Cowan, 1993; Liao et al., 1998; Tripathy et al., 2018). For a comprehensive overview of MAP subtypes and functions, we recommend the following review (Bodakuntla et al., 2019).

With this plethora of functions, it is not surprising that each family of MAPs adopts a unique structural conformation and interacts with microtubules differently (Amos and Schlieper, 2005; Bodakuntla et al., 2019). There is no consensus amino acid sequence or 3D structure for the microtubule-binding domain of different MAP families. In fact, some of the domains reported assume distinct forms; either helical coiled-coils or hairpins, or more globular domains like the CAP-Gly and calponin-homology domain found in end-binding MAPs (Amos and Schlieper, 2005). These observations highlight the potential for several MAPs to decorate microtubules simultaneously. For example, doublecortin (DCX) which is a neuronal MAP, is known to bind adjacent protofilaments, providing a contact point between protofilaments (Bechstedt and Brouhard, 2012). On the other hand, tau, one of the first MAPs to be identified, binds to the microtubule surface, longitudinally along protofilaments (Amos and Schlieper, 2005). Moreover, it has been demonstrated that specific tubulin PTMs affect the interaction with several MAPs, such as the regulation of Tau binding through polyglutamylation of tubulin CTTs (Boucher et al., 1994; Gadadhar et al., 2017; Bodakuntla et al., 2019; Hausrat et al., 2022).

### The tubulinopathies

**FIGURE 1:**
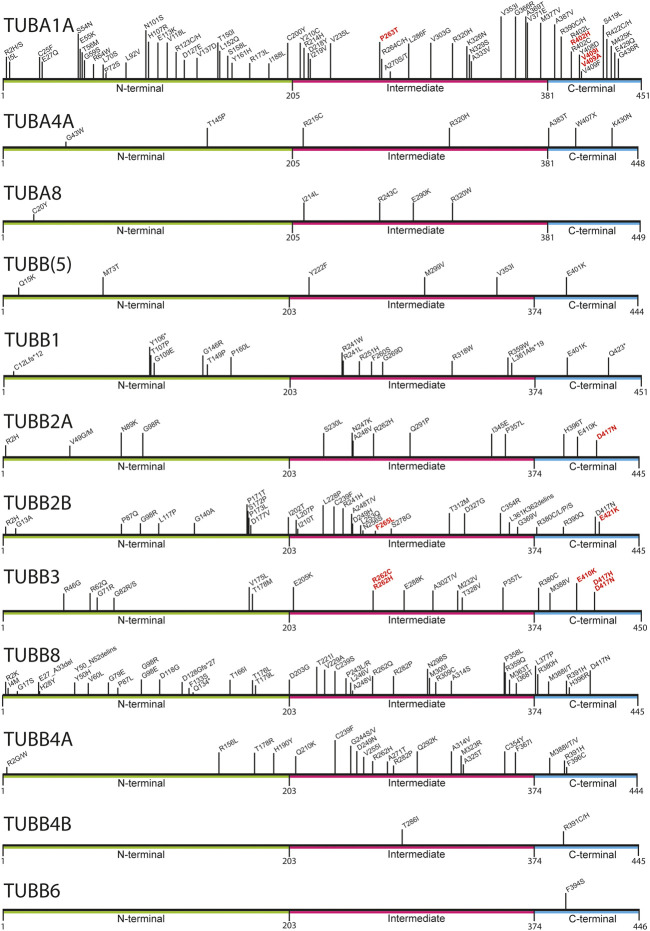
Mapping of pathogenic tubulin mutations across tubulin isotypes. Tubulin proteins can be divided into three regions: the N-terminal (green), Intermediate (pink), and C-terminal (blue) domains. The latter constitutes the major MAP-binding region of tubulin. Variants shown to affect MAP interaction are highlighted in red.

Mutations in multiple tubulin genes have been associated with human disease. Known collectively as the ‘tubulinopathies’, this disease spectrum encompasses numerous neurodevelopmental disorders including, microcephaly, lissencephaly, and polymicrogyria, reflecting the large number of tubulin genes expressed during embryonic brain formation (e.g., *TUBA1A*, *TUBB2A*, *TUBB2B*, *TUBB3*, *TUBB5*) (Keays et al., 2007; Jaglin et al., 2009; Poirier et al., 2010; Breuss et al., 2012; Cushion et al., 2014; Romaniello et al., 2018). In addition to cortical malformations, the tubulinopathies also include disorders of ocular motor function (associated with variants in *TUBB3*, *TUBB2B* & *TUBA1A*), whispering dysphonia (*TUBB4A*), amyotrophic lateral sclerosis (*TUBA4A*), female meiotic infertility (*TUBB8*), Leber congenital amaurosis with hearing loss (*TUBB4B*), and macrothrombocytopaenia (*TUBB1*) (Kunishima et al., 2009; Bahi-Buisson et al., 2014; Smith et al., 2014; Feng et al., 2016; Luscan et al., 2017; Strassel et al., 2019; Jurgens et al., 2021; Kimmerlin et al., 2022). Irrespective of the clinical attributes of the disease the tubulinopathies are predominantly due to *de novo* heterozygous, missense mutations and are predicted to act in a gain-of-function manner in most instances (Figure 1) (Romaniello et al., 2018; Leca et al., 2020). The different diseases that result from tubulin mutations is thought to reflect the expression pattern of a given isoform and the functional repertoire of that protein. Nevertheless, mutations in different tubulin genes can result in strikingly similar phenotypes. For instance, a E421K mutation in TUBB2B and a R262C mutation in TUBB3 both cause ocular motor dysfunction, whereas individuals with a T312M variant in TUBB2B and a R46G mutation in TUBB3 both present with multifocal polymicrogyria (Jaglin et al., 2009; Tischfield et al., 2010; Cederquist et al., 2012; Bahi-Buisson et al., 2014). This raises the prospect that a critical determinant that predicts a disease outcome is the actual amino acid mutated, and the molecular pathway disrupted.

**FIGURE 2:**
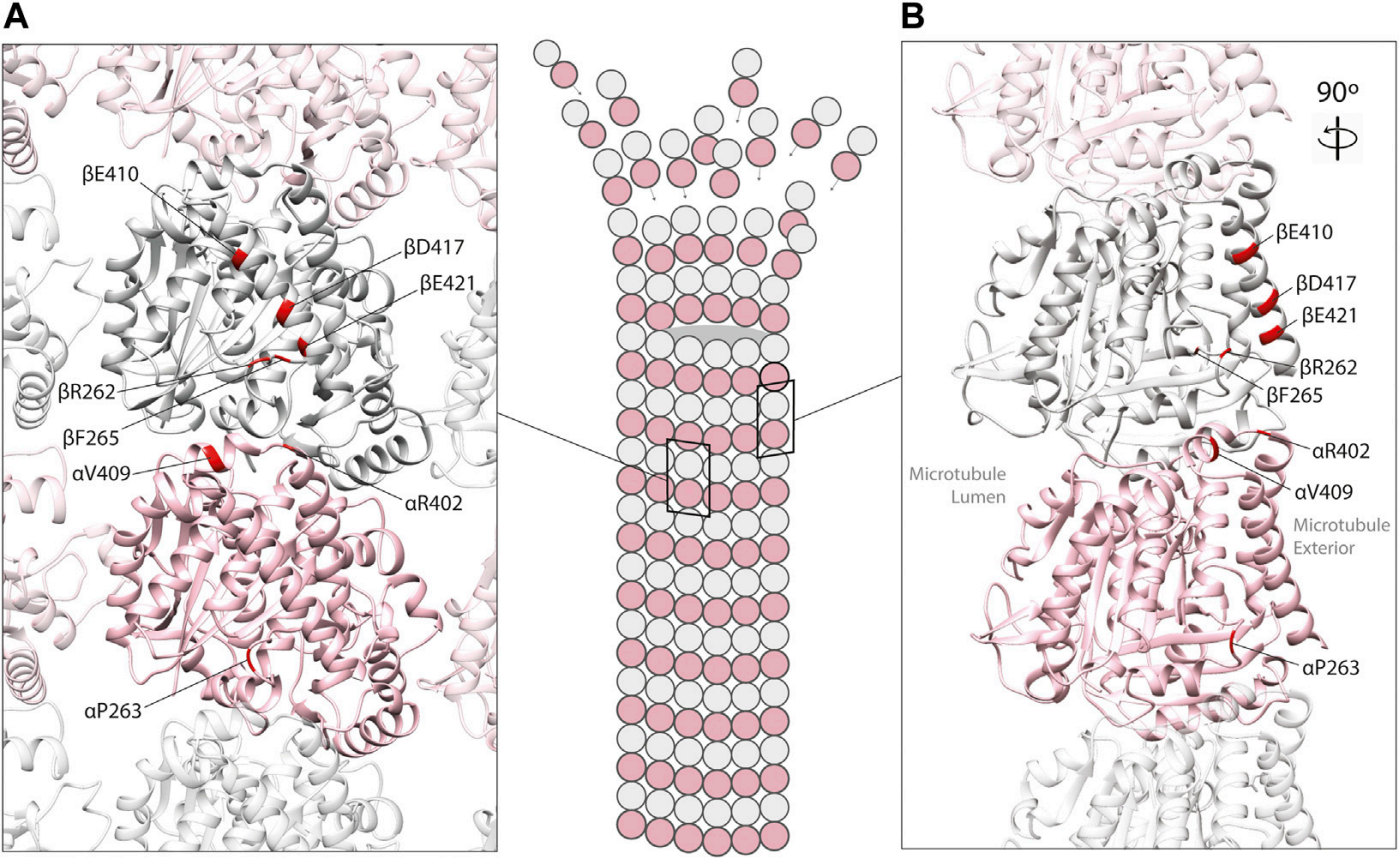
*In silico* homology model of an α/β-tubulin heterodimer **(A)** facing the exterior surface of a microtubule polymer and **(B)** a rotated side view of an individual microtubule protofilament (PDB 2XRP) (Fourniol et al., 2010). α-tubulin is represented by a pink ribbon, β-tubulin in silver. Unstructured, tubulin carboxy-terminal tails are not shown. Mutated residues shown to effect MAP binding are mapped onto α- and β-tubulin subunits (red).

Mechanistic studies have explored how tubulin mutations can influence microtubule biology. Some variants have been shown to perturb the chaperone-mediated folding of heterodimers (e.g., TUBA1A L397P) (Tian et al., 2010), while others have no detectable influence on folding whatsoever (e.g., R402H in TUBA1A), generating heterodimers that integrate into microtubules with ease. The latter are of particular interest because recent studies have highlighted that they can alter the interaction between microtubule polymers with MAPs (Figure 2). In this review, we focus on the tubulin gene variants that have been shown to modify binding of MAPs including kinesin, dynein, XMAP215, CLASP and EB1 (Table 1), how these might affect microtubule function, and the extent to which they determine the disease state.

**TABLE 1:** Tubulin gene variants shown to affect MAP interaction. H = alpha-helix; B = beta-strand; CFEOM3 = Congenital Fibrosis of the extraocular muscle; CFEOM3 = Congenital Fibrosis of the extraocular muscle type 3; MCD = Malformations of cortical development.

Kinesin				
*Variant*	*Isotype*	*Subunit Position*	*Patient Phenotype*	*References*
R262C	TUBB3	H8-B7 Loop	Eye movement disorder (CFEOM3)	Tischfield et al. (2010)
R262H	TUBB3	H8-B7 Loop	Eye movement disorder (CFEOM3)	Tischfield et al., 2010, Ti et al. (2016)
E410K	TUBB3	H11-H12 Loop	Eye movement disorder (CFEOM3)	Tischfield et al. (2010)
D417N	TUBB3	H12	Eye movement disorder (CFEOM3)	Tischfield et al. (2010)
D417H	TUBB3	H12	Eye movement disorder (CFEOM3)	Tischfield et al. (2010), Ti et al. (2016)
E421K	TUBB2B	H12	MCD (polymicrogyria) and eye movement disorder (CFEOM)	Cederquist et al. (2012)
Dynein				
R402H	TUBA1A	H11-H12 Loop	MCD (Lissencephaly)	Aiken et al. (2019), Leca et al. (2020)
Bim1 (EB1)				
F265L	TUBB2B	B7	MCD (Polymicrogyria)	Denarier et al. (2019)
XMAP215				
V409I	TUBA1A	H11-H12 Loop	MCD (Pachygyria)	Hoff et al. (2022)
V409A	TUBA1A	H11-H12 Loop	MCD (Agyria)	Hoff et al. (2022)
CLASP1 & CLASP2				
P263T	TUBA1A	H8-B7 Loop	MCD (Lissencephaly)	Yu et al. (2016)
R402H	TUBA1A	H11-H12 Loop	MCD (Lissencephaly)	Yu et al. (2016)

### Motor proteins: Kinesins

Kinesins are one of two major microtubule-associated molecular motors. There are at least 45 mammalian “KIF” genes that can be broadly categorised into two main types: 1) motile kinesins which use ATP chemical energy to shuttle intracellular cargo along microtubule ‘tracks’ (usually towards polymer plus-ends); and 2) non-motile kinesins that de-polymerise microtubules (Dagenbach and Endow, 2004). Motile kinesins (e.g., KIF1A, KIF1Bβ, KIF5A and KIF21A) are particularly important for neuronal function and survival, as essential proteins within axons and synaptic termini must be transported considerable distances from the cell body (Hirokawa et al., 2009). They are also crucial for mitotic division, facilitating efficient chromosomal congression and segregation during cell division (Wordeman, 2010).

**FIGURE 3:**
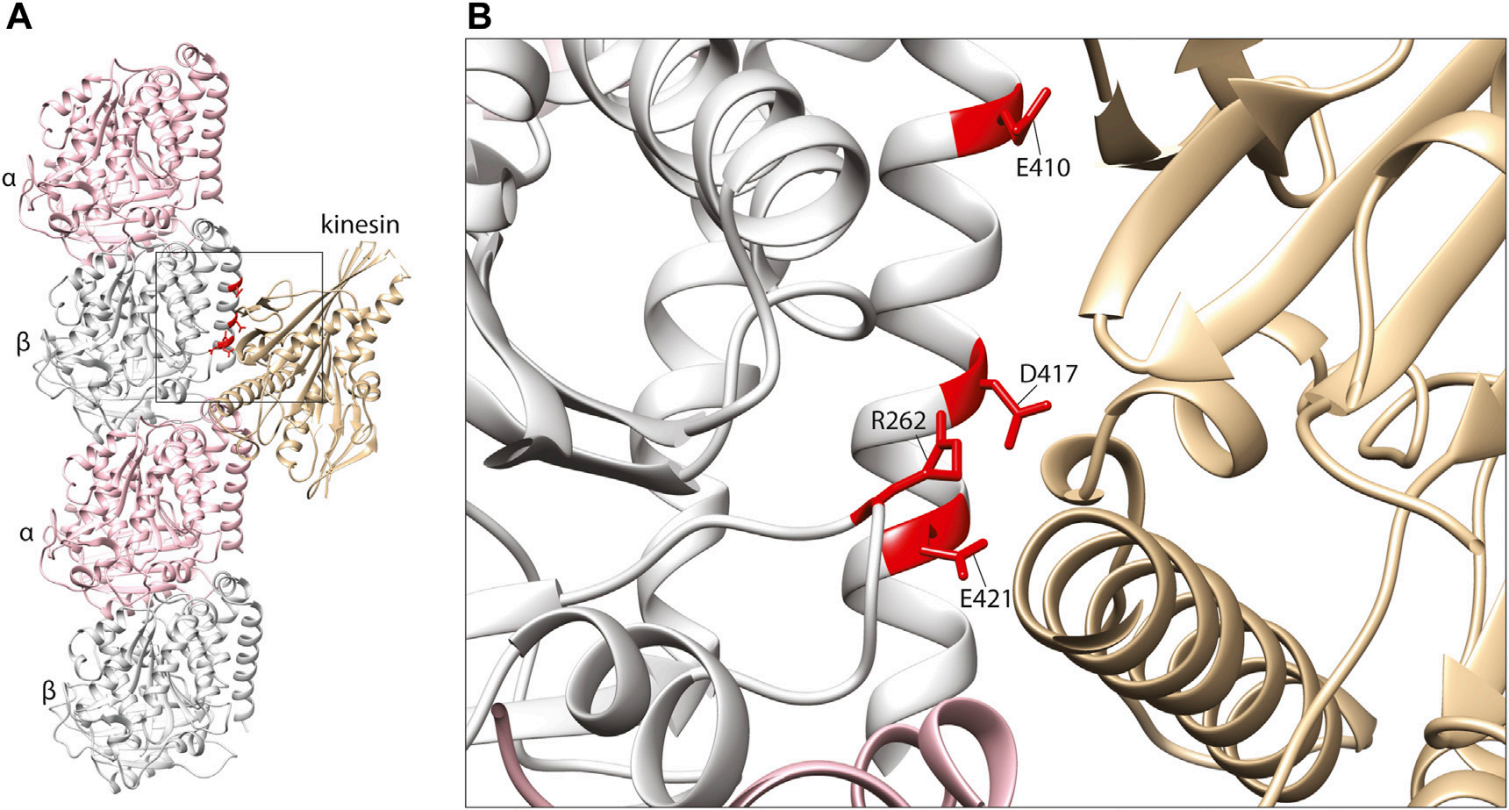
**(A)** *In silico* homology model of a microtubule protofilament with bound kinesin. α-tubulins are represented by a pink ribbon, β-tubulins in silver and kinesin in gold (PDB 6ZPI) (Atherton et al., 2020). **(B)** A detailed view of the predicted kinesin-interacting domain of β-tubulin. Mutated residues shown to effect kinesin binding and/or processivity shown in red.

The key sites of interaction between tubulins and kinesins were initially discovered using alanine mutation scanning, identifying positively charged amino acids on the motor protein corresponding to three negatively charged residues on the β-tubulin subunit: E410, D417, and E421 (Woehlke et al., 1997; Uchimura et al., 2006) (Figure 3). These three amino acids are conserved throughout β-tubulins, and pathogenic mutants affecting these positions have since been identified in TUBB2A, TUBB2B, TUBB3, and TUBB8 (Tischfield et al., 2010; Cederquist et al., 2012; Feng et al., 2016; Sferra et al., 2018). The effects of TUBB3 mutants on kinesin function have been examined comprehensively. TUBB3 is a neuron-specific tubulin isotype (Cleveland, 1987; Joshi and Cleveland, 1989), and *TUBB3* mutations typically cause structural brain malformations and/or congenital fibrosis of the extraocular muscle 3 (CFEOM3), an axon guidance disorder affecting the muscles that control the eye (Poirier et al., 2010; Tischfield et al., 2010). In order to model the disease mechanisms of TUBB3-related CFEOM3, Tischfield and others generated and characterised a TUBB3 R262C mouse mutant (Tischfield et al., 2010). Consistent with patients carrying this variant, the mouse mutant exhibited axon guidance defects (including in the oculomotor nerve), but otherwise normal brain architecture. The authors hypothesised that kinesin dysfunction may be implicated in the pathology of CFEOM3, as mutations in *KIF21A* had been shown to cause similar oculomotor defects (Yamada et al., 2003). Despite not binding directly with kinesin, the R262 residue is predicted to form a H-bond with D417 (Figure 3B) (Tischfield et al., 2010). Co-immunoprecipitation of brain lysates revealed a reduction of microtubule-bound KIF21 in R262C mutant mice compared to wild-type littermates, suggesting that loss of the R262-D417 H-bond alters the tertiary structure of β-tubulin at the kinesin-interacting interface. To test their hypothesis on a wider range of TUBB3 variants, the authors turned to a budding yeast system to assess kinesin processivity. Using this model, they assessed the accumulation of yeast kinesins, Kip3p & Kip2p, at the growing tip of microtubules (Carvalho et al., 2004; Gupta et al., 2006). Compared to wild-type controls, yeast expressing TUBB3 R262C, R262H, E410K, D417H & D417N showed a significant reduction of kinesin at microtubule plus ends, further implicating the motor protein in the disease state (Tischfield et al., 2010).

Building upon these findings, Hirokawa and others investigated fourteen β-tubulin mutations associated with either CFEOM3 or brain malformations using overexpression in dissociated mouse neurons (Niwa et al., 2013). Tracking known kinesin cargos, VAMP2, RAB3, and mitochondria, they observed diminished axonal transport in the presence of the TUBB3 mutants E410K and D417H (Nangaku et al., 1994; Tanaka et al., 1998; Niwa et al., 2008; Song et al., 2009; Niwa et al., 2013). Microtubule co-sedimentation confirmed a reduction in kinesin binding with these two variants, with significantly increased levels of kinesin (but not dynein) detected in the cytoplasmic fraction (Niwa et al., 2013). Importantly, variants associated with only mild CFEOM3 or cortical malformations exhibited normal kinesin function in these assays, supporting a potential phenotype-specific disease pathway. Consistent with a gain-of-function mechanism, the authors showed that mutant subunits only perturb kinesin function when incorporated into microtubules (Niwa et al., 2013). Furthermore, such effects were not restricted to TUBB3, as axonal transport defects were also observed for equivalent substitutions in TUBB2B and TUBB5 (Niwa et al., 2013). This observation was corroborated by reduced kinesin binding and processivity due to two further gene variants, TUBB2A D417N and TUBB2B E421K, associated with progressive neuropathy and CFEOM, respectively (Cederquist et al., 2012; Sferra et al., 2018). These phenotypes are distinct from the cortical brain malformations which are commonly associated with genetic changes in *TUBB2A* and *TUBB2B* (Romaniello et al., 2018). Taken together, these data highlight the existence of mutation specific disease mechanisms that diminish kinesin interaction and intracellular trafficking necessary to generate, guide and maintain healthy and functional neuronal processes.

### Motor proteins: Dynein

The second major family of microtubule-bound motor proteins are the dyneins. In a similar fashion to kinesins, dyneins shuttle along microtubule ‘tracks’ using energy generated through ATP hydrolysis. They are large complexes composed of two identical heavy chains which include the microtubule binding domain and a number of intermediate and light chains (Vallee et al., 2004). They are responsible for transport of intracellular cargo towards microtubule minus ends and are involved in both cell division and cell migration (Vale, 2003).

**FIGURE 4:**
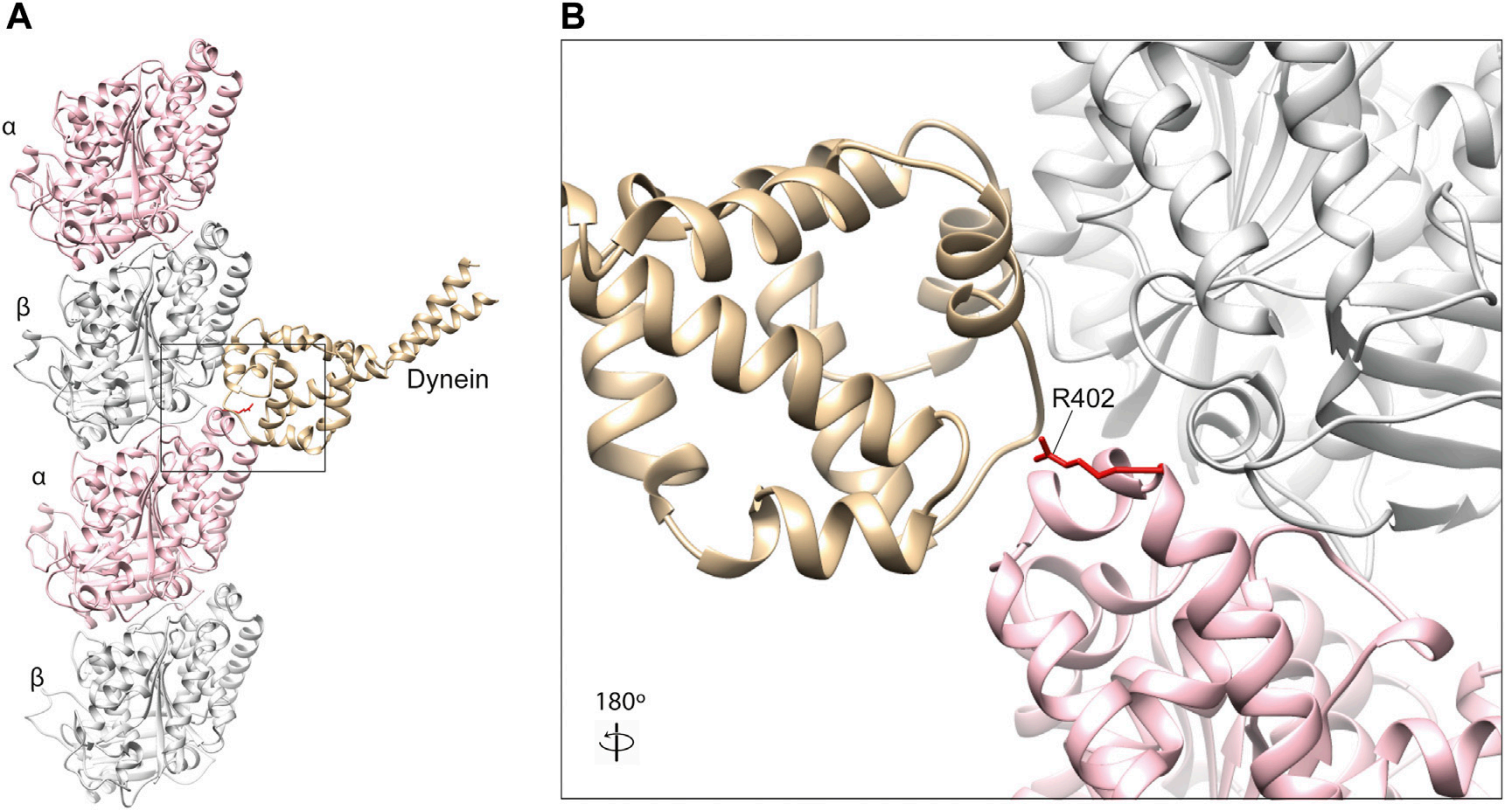
**(A)** *In silico* homology model of a microtubule protofilament with bound dynein. α-tubulins are represented by a pink ribbon, β-tubulins in silver and dynein microtubule-binding domain in gold (PDB 3J1T) (Redwine et al., 2012). **(B)** A detailed view of the dynein-interacting domain. The R402 residue of α-tubulin (red) is a hot-spot for pathogenic variants in TUBA1A, which have been shown to affect dynein interaction and processivity (Aiken et al., 2019; Leca et al., 2020).

Key dynein-binding tubulin residues were first probed for using a mutant alanine screen (Uchimura et al., 2015). This highlighted two α-tubulin residues of particularly importance, R402 (Figure 4) and E415, located within the H11–H12 loop and alpha-helix 12 of the α-tubulin subunit respectively (Nogales et al., 1998; Uchimura et al., 2015). These amino acids are predicted to form salt bridges with one another stabilising the α-tubulin C-terminal hairpin structure important for MAP binding, as well as forming salt bridges directly with dynein (Lowe et al., 2001; Aiken et al., 2019). Despite this, binding between microtubules and the motor protein was still observed even after substituting these residues to alanine (Uchimura et al., 2015). Directional movement was completely ablated however, suggesting R402 and E415 function primarily as structural signals for ATPase activation (Uchimura et al., 2015).

To our knowledge, pathogenic α-tubulin mutants affecting E415 have not been reported. R402, on the other hand, is a known hot-spot for human mutations in *TUBA1A,* with substitutions of this residue constituting almost a third of all reported variants in the gene (Bahi-Buisson et al., 2014). Individuals with R402 C/H/L substitutions commonly present with lissencephaly, a severe cerebral cortex malformation caused by defective neuronal migration during brain development (Moon and Wynshaw-Boris, 2013). Consistent with this observation mutations in dynein heavy chain (DYNC1H1) also cause cortical malformations (Vissers et al., 2010; Poirier et al., 2013), as do variants affecting dynein regulators LIS1 and NDEL1 (Yamada et al., 2008).

Using a yeast as a model system, Aiken and others investigated the consequences of two TUBA1A arginine 402 variants (R402C and R402H) on dynein function (Aiken et al., 2019). They generated analogous mutants in *Saccharomyces cerevisiae*, substituting the equivalent arginine (R403) in the major yeast α-tubulin, Tub1. They confirmed that both mutants form functional tubulin heterodimers which incorporate into endogenous microtubules, supporting a gain-of-function mechanism (Aiken et al., 2019). Unlike the multiple specialised roles of dynein in neurons, dynein’s solitary task in yeast is to translocate the nucleus and mitotic spindle across the plane of cytokinesis (Aiken et al., 2019). Aiken and others analysed hydroxyurea-induced S-phase arrest to isolate spindle sliding events, providing a clear and robust readout to assess mutant effects on dynein function. They reported a reduction in frequency and distance of sliding events for both R403C&H mutants compared to wild type. Importantly, they confirmed dynein recruitment to microtubule plus-ends (a prerequisite for retrograde locomotion) was normal and that kinesin function was undisturbed, suggesting these mutants directly affect dynein interaction and processivity (Aiken et al., 2019).

These conclusions have been further supported by an independent study in our lab which generated a conditional *Tuba1a* R402H mutant mouse (Leca et al., 2020). Expression of the R402H mutation in both the developing cortex and hippocampus resulted in a severe defect in neuronal migration, consistent with the patient phenotype. To gain insight into the underlying molecular mechanisms we performed microtubule co-sedimentation on brain lysates and undertook quantitative mass spectrometry. Comparison of the “microtubule proteome” identified 286 proteins that were significantly altered in R402H animals, seven of which were known MAPs (Leca et al., 2020). Western blot analysis of these seven proteins confirmed that five were present at lower levels in brain lysates R402H mutants (VAPA, VAPAB, REEP1, EZRIN, and PRNP). Only dynein intermediate chain (DYNC1I1) was expressed at endogenous levels but associated less with microtubules. To assess dynein processivity in the presence of this variant, cortical neurons were cultured from mutant and wild-type mice and live cell tracking of dynein-mediated lysosomal transport performed. This highlighted a significant reduction in lysosomal run length, suggesting that dynein processivity towards the cell soma was compromised in mutant animals (Leca et al., 2020). Consistent with dynein dysfunction, we showed a defect in nucleus-centrosome coupling in R402H animals indicative of impaired dynein-mediated nucleokinesis and migration (Leca et al., 2020). Intriguingly, we did not observe any difference in the levels of sedimented dynein heavy chain (DYNC1H1) which binds directly to microtubules, in contrast to the intermediate chain (DYNC1I1) which serves as a bridge between the heavy chain and cargo adaptor (Carter et al., 2008; Schroeder et al., 2014). Moreover, given the large number of proteins dysregulated in R402H animals, it is apparent that a single point mutation can have pleiotropic effects, potentially on multiple uncharacterised MAPs.

### Microtubule plus-end MAPs (and TAPs)

Microtubule plus-end MAPs or “+TIPs” collectively describe a diverse subset of proteins which, as their name may suggest, localize at the growing tips of microtubule polymers (Perez et al., 1999; Mimori-Kiyosue et al., 2000; Jiang et al., 2012). Certain plus-end MAPs, such as the end-binding protein family (EB1, EB3) are known to recruit and form complex networks with other + TIPs at this region of the microtubule polymer, whereas others bind to the microtubule end directly (Honnappa et al., 2005; Slep et al., 2005; Honnappa et al., 2009; Al-Bassam et al., 2010; Kumar et al., 2017). Plus-end MAPs generally regulate one or more of the basic parameters underlying microtubule dynamics: rate of polymerisation, rate of de-polymerisation, the frequency of catastrophe and/or the frequency of rescue (Straube, 2011). Examples include microtubule polymerases (e.g., XMAP215s) that catalyse and accelerate microtubule growth, and Cytoplasmic Linker-Associated Proteins (CLASPs) that stem microtubule depolymerisation events and potentiate re-growth (Gard and Kirschner, 1987; Al-Bassam and Chang, 2011; Moriwaki and Goshima, 2016). Evidence suggests that tubulin mutations can affect the correct localisation and function of these specialised MAPs, further highlighting their critical role in microtubule biology.

### Plus-end MAPs: XMAP215 family microtubule polymerases

**FIGURE 5:**
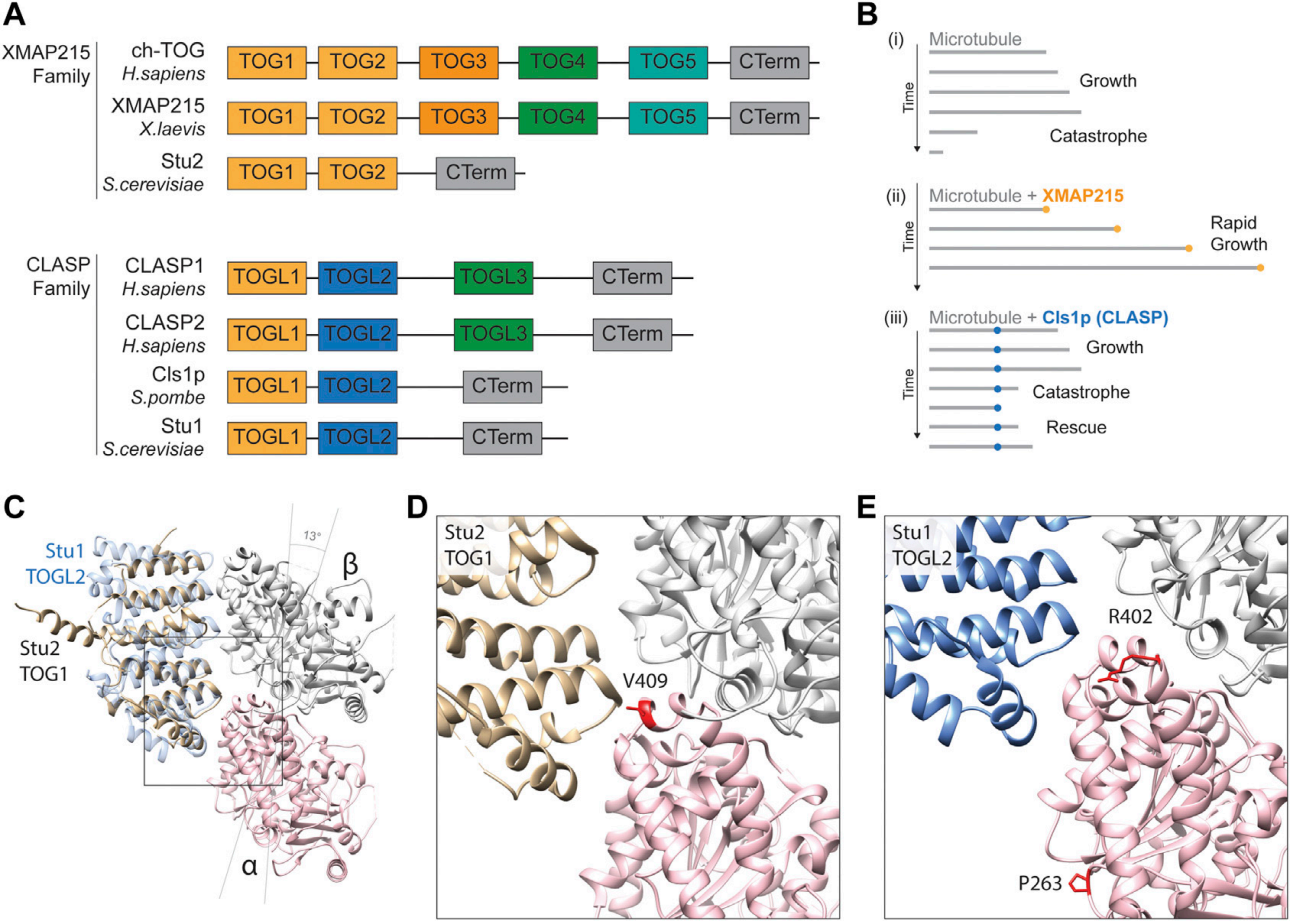
**(A)** Schematic alignment and positional conservation of TOG domains in XMAP215 family microtubule polymerases and CLASPs [adapted from (Al-Bassam and Chang, 2011; Byrnes and Slep, 2017)]. **(B)** Schematic depiction of dynamic behaviour of (i) pure microtubules (grey lines), (ii) accelerated microtubule polymerisation in the presence of XMAP215 family polymerases (gold), and (iii) microtubule rescue mediated by fission yeast CLASP, Cls1p (blue), adapted from (Al-Bassam and Chang, 2011). **(C)** Homology model depiction of a unpolymerised tubulin heterodimer complexed with TOG1 domain of *S. cerevisiae* microtubule polymerase Stu2 (gold) (PDB 4FFB) and docked TOGL2 domain of Stu1 (*S. cerevisiae* CLASP; blue) (PDB 6COK) (Ayaz et al., 2012; Majumdar et al., 2018). α-tubulins are represented by a pink ribbon, β-tubulins in silver. Both TOGs bind preferentially to curved tubulin heterodimers, hence α- and β-tubulin subunits are tilted 13 to form this complex (Ayaz et al., 2012). **(D)** A detailed view of the predicted TOG1 binding interface of tubulin heterodimers. The α-tubulin residue valine 409 (410 in yeast) (red) is directly involved in TOG1 binding, with substitutions affecting its binding and localisation to microtubule plus-ends (Hoff et al., 2022). **(E)** Detailed view of the predicted TOGL2 binding interface of tubulin heterodimers. Mutating α-tubulin proline 263 and arginine 402 (P264 and R402 in yeast) have been shown to reduce binding affinity with human CLASPs 1 & 2 (Yu et al., 2016).

This family is named after the XMAP215 microtubule polymerase identified in *Xenopus laevis* but also comprises human Colonic and Hepatic Tumour Overexpressed Gene (ch-TOG), Minispindles (*D. melanogaster*), and Stu2 (*S. cerevisiae*) (Gard and Kirschner, 1987; Wang and Huffaker, 1997; Charrasse et al., 1998; Cullen et al., 1999). As with many plus end-binding proteins, the XMAP215s are composed of arrayed tubulin-binding Tumour Overexpressed Gene (TOG) motifs, containing 250 amino acid residue repeats (Akhmanova et al., 2001; Cassimeris et al., 2001; Leano et al., 2013). Two of these, TOG1 and TOG2, are structurally conserved and present in all family members but, in higher eukaryotes, five TOG domains are separated by unstructured linkers of 60–100 residues (Figure 5A) (Currie et al., 2011; Widlund et al., 2011). The TOG1 and TOG2 domains of yeast Stu2 and their association with tubulin have been examined in detail (Ayaz et al., 2012). TOG1 and TOG2 are predicted to bind tubulins at a 1:1 ratio, but with a particular affinity for curved heterodimer conformations. This propensity for curved tubulin localises these polymerases to the growing tip as, here, recently incorporated tubulins initially assume an expanded and flayed configuration before integrating into the microtubule lattice (Ayaz et al., 2014).

Microtubule polymerisation is highly dependent on local concentrations of un-polymerised “free” tubulin surrounding the growing tip (Desai and Mitchison, 1997; Howard and Hyman, 2009). Through their tubulin-binding TOG domains, XMAP215s enrich local concentrations of free tubulin near microtubule plus-end (Figure 5B). TOG domains are thought to work in a coordinated fashion to catalyse polymerisation, as they can discriminate between conformational states of tubulin dimers (Ayaz et al., 2012). Initially, TOG1 recognises and captures naturally curved free heterodimers, subsequently recruiting them into a growing microtubule. Upon integration into the microtubule, heterodimers assume a slightly straighter configuration, after which they can no longer bind TOG1 but are “handed-off” to TOG2. When fully embedded into the microtubule lattice formation, the conformation of tubulin becomes too straight for TOG2, which in turn releases the polymerised heterodimer (Ayaz et al., 2012). It has since been shown that TOGs 1-3 preferentially bind curved heterodimers, whilst TOG 4 and 5 are structurally distinct and bind microtubule-incorporated subunits (Byrnes and Slep, 2017).

A key residue at the heart of the TOG1/2-tubulin interface is valine 409 of α-tubulin, located within the H11-H12 loop (Figures 5C, D) (Nogales et al., 1998; Ayaz et al., 2012). Two pathogenic variants in TUBA1A have been reported at this residue, V409I and V409A, identified in individuals with pachygyria and agyria (mild and severe lissencephaly) respectively (Bahi-Buisson et al., 2014; Fallet-Bianco et al., 2014). Given the position of the residue, Hoff and others sought to investigate whether this mutation altered XMAP215 interaction (Hoff et al., 2022). Mutating the equivalent valine (V410) in yeast α-tubulin Tub1 to isoleucine or alanine they showed that both V409I and V409A mutants diminished the levels of Stu2/XMAP215 at the microtubule tip through a reduction in Stu2 TOG1 binding affinity (Hoff et al., 2022). Surprisingly they showed that this caused an increase in microtubule polymerisation rates, concurrent with a reduction in the frequency of catastrophes (Hoff et al., 2022). The effects on polymerisation speeds were echoed when overexpressing these mutants in mouse primary neurons and, in both models, the effects were more prominent with the valine to alanine mutant, concomitant with the more severe phenotype in the individual carrying the TUBA1A p.V409A mutation (Hoff et al., 2022). In order to reconcile the somewhat contradictory decrease in TOG binding of α-tubulin mutants with faster polymerisation rates, Hoff and others proposed a model by which the V410I&A mutants result in straighter heterodimers that weaken the binding potential of Stu2 TOG domains but, simultaneously, favour efficient microtubule incorporation and subsequent growth (Hoff et al., 2022). Whilst this hypothesis remains to be tested it provides an important reminder that multiple aspects of microtubule function can be perturbed simultaneously by individual tubulin amino acid substitutions.

### Plus-end MAPs: CLASPs

The CLASPs are MAPs that counteract microtubule catastrophe by stabilising de-polymerisation and potentiating ‘rescue’ (Al-Bassam et al., 2010). This family includes human CLASP1 and CLASP2, Stu1 into yeast (*S. cerevisiae*) and Cls1p (or Peg1) in fission yeast (*S. pombe*) (Yin et al., 2002; Grallert et al., 2006; Sousa et al., 2007). Whilst CLASPs are known to associate with microtubule plus-end through EB1-mediated recruitment (Honnappa et al., 2009), they are also able to bind the microtubule polymer lattice directly through two serine/arginine-rich C-terminal domains (Wittmann and Waterman-Storer, 2005; Al-Bassam et al., 2010). Whilst the C-terminal domain attaches to the microtubule lattice, two parallel N-terminal TOG-like (TOGL) domains (related structurally to those found in XMAP215 polymerases) work in tandem to lasso free tubulin heterodimers in the surrounding cytoplasm (Al-Bassam et al., 2010). Through these TOG-like motifs, CLASPs function as a molecular safety net during microtubule catastrophe (Figure 5B), restoring local concentrations of free tubulin dimers to offset rapid rates of disassembly and initiate microtubule rescue.

Recently, tubulin gene mutations have been shown to affect CLASP recognition and/or binding (Yu et al., 2016). To identify novel Tubulin-Associated Proteins (TAPs), Yu and others developed a dual tubulin expression construct system to introduce equimolar levels of *TUBB3* and biotinylated *TUBA1A* into HEK293T cells (Yu et al., 2016). Complementing this with quantitative mass spectrometry, they could identify TAPs pulled down with transgenic tubulin through streptavidin-mediated purification. Among the most abundant proteins in the “Tubulome” were CLASP1 & CLASP2 (Yu et al., 2016), confirming a strong affinity of CLASP TOG-like domains for free tubulin (Al-Bassam et al., 2010). The authors built upon their initial dataset by introducing two tubulin variants associated with human cortical malformations into this expression vector: TUBA1A P263T and R402H (Poirier et al., 2007; Yu et al., 2016). When expressing these mutant constructs in this system, the authors detected notable reductions in the pull-down efficiency of both CLASP1 & CLASP2, as well as the Golgi-associated protein GCC185, a known interactor of CLASP (Efimov et al., 2007; Yu et al., 2016). Interestingly, whilst arginine 402 is near the predicted interface between CLASP TOG-like regions and tubulin (Figures 5C, E), proline 263 looks unlikely to contribute directly to CLASP binding. The TUBA1A P263T variant might therefore affect CLASP binding indirectly through allosteric changes to the tubulin heterodimer.

### Plus-end MAPs: EBs

**FIGURE 6:**
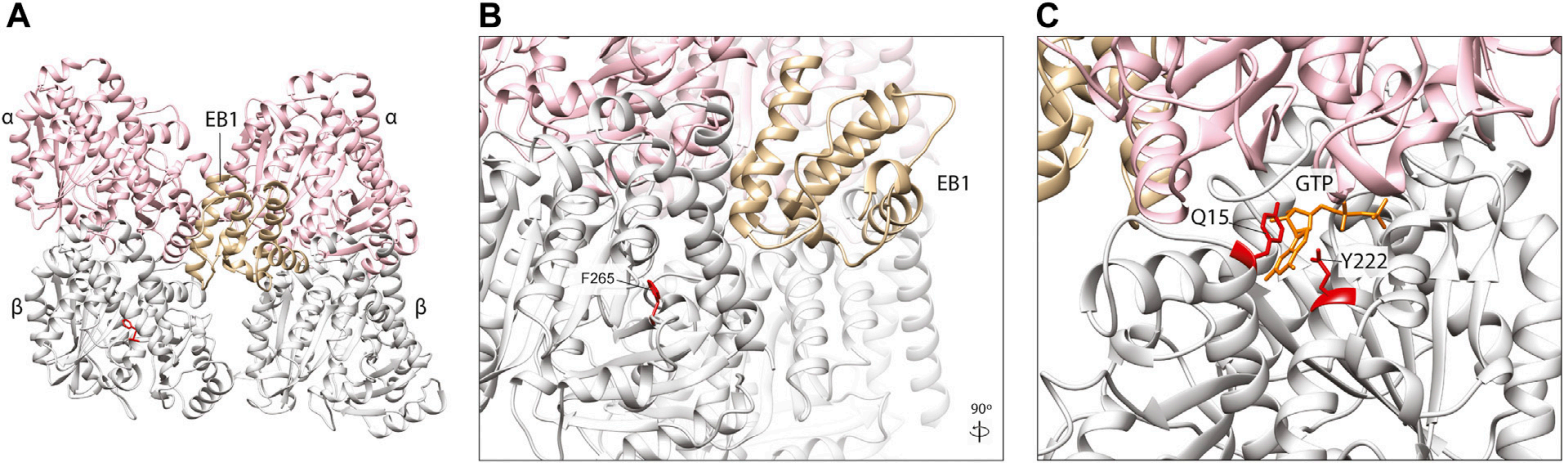
**(A)** *In silico* homology model of a microtubule protofilament with bound Calponin Homology domain of *S. pombe* EB1 homologue, Mal3 (labelled EB1). α-tubulins are represented by a pink ribbon, β-tubulins in silver and EB1 in gold (PDB 4ABO) (Maurer et al., 2012). **(B)** A detailed view of the EB1-tubulin interface. The F265 residue (red), associated with neurodevelopmental disease and shown to affect EB1 binding in yeast (Jaglin et al., 2009; Denarier et al., 2019), is not predicted to interact directly with this plus-end MAP, suggesting it may act *via* allosteric reconfiguration. **(C)**. TUBB5 Q15 and Y222 (red) are located within the β-tubulin GTP (orange)-binding site and variants affecting these residues might to affect nucleotide interaction and/or hydrolysis which, in turn, could influence EB2 binding.

The End-Binding proteins (EB1-3) are an evolutionarily conserved family of plus-end MAPs. They localise to the microtubule tip through an N-terminal Calponin Homology (CH) domain, which binds most efficiently to the newest, stable portion of the microtubule (Hayashi and Ikura, 2003; Maurer et al., 2011). The CH domains bind four tubulin subunits simultaneously, bridging two adjacent protofilaments and at an interdimer interface (between different heterodimers) (Maurer et al., 2012) (Figure 6A). Rather than providing direct structural support, EBs predominantly recruit other plus-end MAPs to this region and are therefore key players in co-ordinating MAP-mediated control of microtubule dynamics (Slep et al., 2005).

A TUBB2B F265L mutation that causes multifocal polymicrogyria (Jaglin et al., 2009), has been shown to disrupt binding of the yeast homologue of EB1 known as Bim1 (Denarier et al., 2019). Denarier and others introduced the F265L mutation into Tub2 in yeast, and demonstrated that it incorporates into microtubules (Denarier et al., 2019), despite previous evidence that it compromises protein folding and heterodimerisation efficiency (Jaglin et al., 2009). This resulted in a viable yeast strain with microtubules that were smaller but more stable, with a reduced frequency of microtubule catastrophe events, increased pause duration and increased resistance to the depolymerising drug Benomyl (Denarier et al., 2019). This was attributed to perturbation of microtubule association with Bim1, the yeast homologue of EB1 which showed a marked reduction in plus end binding in the case of the F265L mutant. Surprisingly, F265 which is located within a beta-sheet (B7) of the β-tubulin subunit (Nogales et al., 1998), does not lie at the key point-of-contact between EB1 and microtubules (Maurer et al., 2012; Howes et al., 2017). This residue sits within the β-tubulin ‘intermediate domain’, and its side chain is angled towards the core of the subunit (Figures 6A, B) where it is potentially involved in maintaining the structural integrity of the globular protein. EB binding, however, is thought to be sensitive to subtle structural changes within the microtubule lattice, closely linked to the nucleotide state of heterodimers at the polymer tip (Maurer et al., 2012). Accordingly, the authors hypothesised that the F265L variant affects EB1/Bim1 interaction indirectly, through conformational changes to the β-tubulin and/or adjacent subunits, which modify the CH binding pocket at the microtubule exterior (Denarier et al., 2019).

Whilst all EB family proteins (EB1-3) bind microtubule-tips through conserved CH domains, they are each thought to bind to spatially distinct sites with preference for different tubulin nucleotide states (Roth et al., 2018). EB2 is arguably the outlier in this protein family; it is the most diverse in terms of amino acid sequence divergence, it does not promote microtubule growth, it does not dimerise, and it associates along the microtubule lattice during mitosis (Juwana et al., 1999; Li et al., 2022). Four EB2-specific microtubule binding residues within the CH domain are thought to contribute to its unique polymer-binding behaviour (Roth et al., 2018). A mutation in one of these residues, R143C, plus two others within the microtubule-binding CH domain (N68S and Y87C) are known to cause Circumferential Skin Creases Kunze Type, a congenital disorder characterised by excessive skin folds, intellectual disability, and dysmorphic features (Isrie et al., 2015). All three variants increase EB2 co-sedimentation with microtubules *in vitro* (Isrie et al., 2015). Intriguingly, two TUBB5 variants, Q15K and Y222C, are also associated with this distinctive condition (Isrie et al., 2015). Given the strong phenotypic overlap between these TUBB5 and EB2 variants, they could act through a common molecular mechanism. Whilst the pathogenic TUBB5 residues are located outside the CH-binding motif, they are proximal to each other within the core of the β-tubulin subunit (Figure 6C). Significantly, they are located within the ‘exchangeable’ GTP binding site, with Q15 known to bind directly to the nucleotide (Lowe et al., 2001). TUBB5 Q15K and Y222C may therefore distort the highly conserved GTP binding motif and/or rate of GTP hydrolysis which could disrupt the nucleotide state-dependent binding of EB2 at microtubule plus-ends (Figure 6C).

## Concluding remarks

To support the diversification of life from simple microbes to complex multicellular organisms, the dynamic behaviour of microtubules has been harnessed to execute a growing assortment of specialised intracellular tasks. To unlock the full potential of these cytoskeletal polymers, complex families of MAPs have co-evolved alongside increasingly diverse tubulin isotypes to finely choreograph distinct populations of microtubule polymers to enable changes in cell morphology, cell migration, intracellular transport, and mitotic division. Mutations in the tubulin genes can have serious consequences for human health, with an ever-expanding array of disease states associated with *de novo* missense mutations. Our challenge is to understand how these mutations cause disease, and to exploit this understanding to develop personalised medicines in the future. This review has focused on a series of emerging studies that have asked how tubulin mutations influence MAP binding, specifically, *KIF21A*, the dynein complex, XMAP215, CLASP1/2, and EB1/2. This analysis has highlighted the importance of mutation specific mechanisms, which perturb a particular pathway and consequentially result in a disease with defined attributes. While some mutations influence MAP binding in a predictable way given their physical proximity, it is evident that tubulin variants can have allosteric effects on microtubule structure thereby influencing MAP binding in unexpected ways. This is perhaps one reason, why the phenotypic prediction of tubulin mutations has proved to be so difficult (Attard et al., 2022).

It is clear that much remains to be discovered. At least 180 tubulin variants have been described in the literature to date, but only twelve (four α- and eight β-tubulin) have been explicitly shown to affect MAP interaction and/or function. This reflects the laborious nature of mechanistic studies in microtubule biology, prompting many investigators to use yeast as a model system. While efficient and robust, yeast cannot replicate the cocktail of MAPs that are present in the mammalian brain, nor the diversity of tubulin isotypes and PTMs. On the other hand it is not feasible or practical to generate conditional mouse models for each tubulin variant. An alternative way forward is to exploit technological developments in stem cell biology and 3D tissue-specific organoid cultures. It is now possible to generate and characterise cerebral organoids from iPSC lines generated from patients with tubulin mutations, alongside CRISPR-repaired isogenic controls. Coupled with advanced quantitative proteomic methods, investigators can interrogate the effect of tubulin mutations on the microtubule proteome in a human system with greater ease (Leca et al., 2020; Rafiei and Schriemer, 2022). This would permit the identification of novel MAPs relevant to disease states, as it likely there are numerous uncharacterised MAPs that have yet to be identified that influence microtubule behaviour in unexpected ways (Jijumon et al., 2022). In the future these cellular systems may provide a powerful and patient-specific platform to screen for personalised therapeutic interventions.
